# Diethyl 2-[(4-nitro­phen­yl)(4-phenyl-1,2,3-selenadiazol-5-yl)meth­yl]malonate

**DOI:** 10.1107/S160053680800706X

**Published:** 2008-03-20

**Authors:** A. Marx, S. Saravanan, S. Muthusubramanian, V. Manivannan, Nigam P. Rath

**Affiliations:** a Department of Physics, Presidency College, Chennai 600 005, India; b Department of Organic Chemistry, School of Chemistry, Madurai Kamarajar University, Madurai 625 021, India; c Department of Chemistry and Biochemistry, University of Missouri–St Louis, 8001 Natural Bridge Road, St Louis, MO 63121, USA

## Abstract

In the title compound, C_22_H_21_N_3_O_6_Se, the heterocyclic ring makes dihedral angles of 50.03 (11) and 67.75 (11)°, respectively, with the benzene and phenyl rings. The terminal C atoms of the ester groups are disordered over two positions: the site occupancies for the C atoms are 0.62 (3)/0.38 (3) and 0.48 (3)/0.52 (3). In the crystal structure, weak intra- and inter­molecular C—H⋯O inter­actions are observed.

## Related literature

For biological activities, see: El-Kashef *et al.* (1986 ▶); El-Bahaie *et al.* (1990 ▶). For closely related compounds, see: Bertini *et al.* (1984 ▶); Gunasekaran *et al.* (2007 ▶).
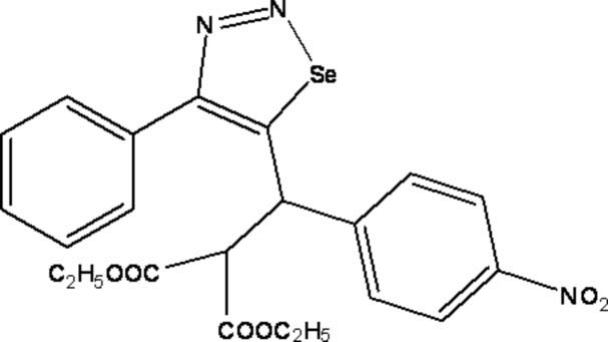


## Experimental

### 

#### Crystal data

C_22_H_21_N_3_O_6_Se
*M* *_r_* = 502.38
Triclinic, 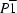

*a* = 9.9530 (6) Å
*b* = 10.5220 (6) Å
*c* = 12.2305 (7) Å
α = 79.350 (3)°
β = 74.632 (3)°
γ = 67.632 (3)°
*V* = 1137.05 (11) Å^3^
*Z* = 2
Mo *K*α radiation
μ = 1.69 mm^−1^
*T* = 295 (2) K
0.23 × 0.17 × 0.16 mm

#### Data collection

Bruker APEXII area-detector diffractometer
Absorption correction: multi-scan (**SADABS**; Sheldrick, 1996 ▶) *T*_min_ = 0.697, *T*_max_ = 0.773
30194 measured reflections
4477 independent reflections
3053 reflections with *I* > 2σ(*I*)
*R*_int_ = 0.028

#### Refinement

*R*[*F* ^2^ > 2σ(*F* ^2^)] = 0.053
*wR*(*F* ^2^) = 0.161
*S* = 1.04
4477 reflections
313 parameters
4 restraints
H-atom parameters constrained
Δρ_max_ = 0.93 e Å^−3^
Δρ_min_ = −0.80 e Å^−3^

### 

Data collection: *APEX2* (Bruker, 2004 ▶); cell refinement: *APEX2*; data reduction: *APEX2*; program(s) used to solve structure: *SHELXS97* (Sheldrick, 2008 ▶); program(s) used to refine structure: *SHELXL97* (Sheldrick, 2008 ▶); molecular graphics: *PLATON* (Spek, 2003 ▶); software used to prepare material for publication: *SHELXL97*.

## Supplementary Material

Crystal structure: contains datablocks global, I. DOI: 10.1107/S160053680800706X/is2278sup1.cif

Structure factors: contains datablocks I. DOI: 10.1107/S160053680800706X/is2278Isup2.hkl

Additional supplementary materials:  crystallographic information; 3D view; checkCIF report

## Figures and Tables

**Table 1:** Hydrogen-bond geometry (Å, °)

*D*—H⋯*A*	*D*—H	H⋯*A*	*D*⋯*A*	*D*—H⋯*A*
C22*A*—H22*D*⋯O2^i^	0.96	2.47	3.38 (4)	159
C11—H11⋯O6^ii^	0.93	2.56	3.410 (6)	152
C18—H18*A*⋯O4	0.97	2.29	2.686 (7)	103
C21—H21*C*⋯O6	0.97	2.17	2.627 (6)	107

Symmetry codes: (i) 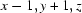; (ii) 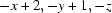.

**Fig. 1.**
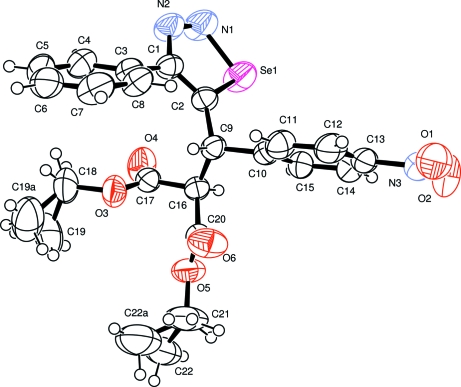
The molecular structure of (I), with atom labels and 50% probability displacement ellipsoids for non-H atoms.

**Fig. 2.**
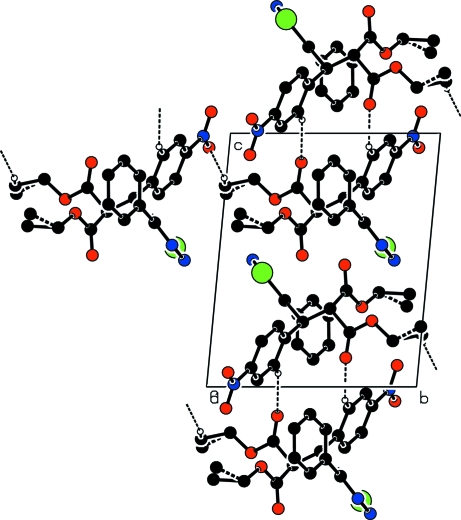
The packing of (I), viewed down the a axis. Hydrogen bonds are shown as dashed lines. H atoms not involved in hydrogen bonds have been omitted.

## supplementary crystallographic information

### Comment

Selenium containing compounds like 1,2,3-selenadiazole possess various beneficial activities like antibacterial (El-Kashef *et al.*, 1986), antimicrobial (El-Bahaie *et al.*, 1990) activities. The geometric parameters in the compound, (I) (Fig. 1), agree with the reported values of similar structures (Bertini *et al.*, 1984; Gunasekaran *et al.*, 2007).

The heterocyclic ring makes the dihedral angles of 50.03 (11) and 67.75 (11)° with the benzene and phenyl rings. The terminal C atoms of the ester groups are disordered over two positions; the site occupancies for the C atoms are 0.62/0.38 and 0.48/0.52. The molecular structure is stabilized by weak intramolecular C—H···O interactions and the crystal packing of (I) is stabilized by weak intermolecular C—H···O contacts (Table 1 and Fig. 2).

### Experimental

2.22 g (0.02 mol) of powdered selenium dioxide was dissolved in glacial acetic acid by gentle warming. To this warm solution, 0.002 mol of diethyl 2-{3-[2-(aminocarbonyl)hydrazono]-1-(4-nitrophenyl)-3-phenylpropyl} malonate was added at once and the mixture was gently heated on a water bath until gas evolution ceased. The selenium deposited on cooling was removed by filtration and the filtrate poured into crushed ice, extracted with chloroform and purified by column chromatography, using silica gel (60–120 mesh) to yield diethyl 2-[(4-nitrophenyl) (4-phenyl-1,2,3-selenadiazol-5-yl)methyl]malonate. Solvent used for crystallization is ethanol.

### Refinement

The site occupancy factors were refined as C19 = 0.62 (3), C19A = 0.38 (3), C22 = 0.48 (3) and C22A = 0.52 (3) during anisotropic refinement. The C21—C22A, C21—C22, C18—C19 and C18—C19A bond distances were restrained to be 1.5 (1) Å. H atoms were positioned geometrically and refined using riding model with C—H = 0.93 Å and *U*_iso_(H) = 1.2*U*_eq_(C) for aromatic C—H, C—H = 0.98 Å and *U*_iso_(H) = 1.2*U*_eq_(C) for CH, C—H = 0.97 Å and *U*_iso_(H) = 1.2*U*_eq_(C) for CH_2_, and C—H = 0.96 Å and *U*_iso_(H) = 1.5*U*_eq_(C) for CH_3_.

### Crystal data

**Table:**

C_22_H_21_N_3_O_6_Se	*Z* = 2
*M**_r_* = 502.38	*F*_000_ = 512
Triclinic, *P*1	*D*_x_ = 1.467 Mg m^−^^3^
Hall symbol: -P 1	Mo *K*α radiation λ = 0.71073 Å
*a* = 9.9530 (6) Å	Cell parameters from 8446 reflections
*b* = 10.5220 (6) Å	θ = 1.7–26º
*c* = 12.2305 (7) Å	µ = 1.69 mm^−^^1^
α = 79.350 (3)º	*T* = 295 (2) K
β = 74.632 (3)º	Block, colourless
γ = 67.632 (3)º	0.23 × 0.17 × 0.16 mm
*V* = 1137.05 (11) Å^3^	

### Data collection

**Table:**

Bruker SMART APEX area-detector diffractometer	4477 independent reflections
Radiation source: fine-focus sealed tube	3053 reflections with *I* > 2σ(*I*)
Monochromator: graphite	*R*_int_ = 0.028
*T* = 295(2) K	θ_max_ = 26.1º
ω and φ scans	θ_min_ = 1.7º
Absorption correction: multi-scan(*SADABS*; Sheldrick, 1996)	*h* = −12→12
*T*_min_ = 0.697, *T*_max_ = 0.773	*k* = −12→12
30194 measured reflections	*l* = −15→15

### Refinement

**Table:**

Refinement on *F*^2^	Secondary atom site location: difference Fourier map
Least-squares matrix: full	Hydrogen site location: inferred from neighbouring sites
*R*[*F*^2^ > 2σ(*F*^2^)] = 0.054	H-atom parameters constrained
*wR*(*F*^2^) = 0.161	*w* = 1/[σ^2^(*F*_o_^2^) + (0.0776*P*)^2^ + 0.9842*P*] where *P* = (*F*_o_^2^ + 2*F*_c_^2^)/3
*S* = 1.04	(Δ/σ)_max_ < 0.001
4477 reflections	Δρ_max_ = 0.93 e Å^−^^3^
313 parameters	Δρ_min_ = −0.80 e Å^−^^3^
4 restraints	Extinction correction: none
Primary atom site location: structure-invariant direct methods	

### Fractional atomic coordinates and isotropic or equivalent isotropic displacement parameters (Å^2^)

**Table:**

	*x*	*y*	*z*	*U*_iso_*/*U*_eq_	Occ. (<1)
Se1	1.00900 (5)	0.21345 (6)	0.44996 (4)	0.0888 (3)	
O1	1.5749 (5)	0.1019 (4)	−0.0847 (4)	0.1160 (15)	
O2	1.6666 (5)	0.0959 (5)	0.0564 (5)	0.1327 (17)	
O4	0.7916 (3)	0.6035 (4)	0.4808 (2)	0.0859 (10)	
O6	1.0425 (4)	0.6499 (3)	0.1149 (2)	0.0859 (10)	
N1	0.8397 (5)	0.1628 (5)	0.5006 (4)	0.0939 (13)	
N2	0.7447 (4)	0.2286 (4)	0.4410 (3)	0.0753 (10)	
N3	1.5628 (6)	0.1283 (4)	0.0109 (5)	0.0929 (14)	
C1	0.7808 (4)	0.3215 (4)	0.3527 (3)	0.0558 (9)	
C2	0.9155 (4)	0.3332 (4)	0.3410 (3)	0.0521 (8)	
C3	0.6670 (4)	0.4007 (4)	0.2854 (3)	0.0542 (9)	
C4	0.5213 (5)	0.4674 (5)	0.3410 (4)	0.0686 (11)	
H4	0.4952	0.4563	0.4201	0.082*	
C5	0.4156 (5)	0.5494 (5)	0.2804 (5)	0.0807 (13)	
H5	0.3193	0.5953	0.3187	0.097*	
C6	0.4514 (6)	0.5637 (5)	0.1641 (5)	0.0837 (14)	
H6	0.3801	0.6198	0.1231	0.100*	
C7	0.5930 (6)	0.4949 (5)	0.1085 (4)	0.0796 (13)	
H7	0.6168	0.5031	0.0293	0.095*	
C8	0.7013 (5)	0.4133 (5)	0.1679 (3)	0.0640 (10)	
H8	0.7970	0.3671	0.1288	0.077*	
C9	0.9818 (4)	0.4288 (4)	0.2556 (3)	0.0495 (8)	
H9	0.9199	0.4661	0.1987	0.059*	
C10	1.1371 (4)	0.3504 (4)	0.1923 (3)	0.0509 (8)	
C11	1.1559 (5)	0.3109 (4)	0.0853 (3)	0.0627 (10)	
H11	1.0739	0.3346	0.0529	0.075*	
C12	1.2940 (5)	0.2375 (5)	0.0274 (4)	0.0738 (12)	
H12	1.3063	0.2109	−0.0441	0.089*	
C13	1.4136 (5)	0.2034 (4)	0.0754 (4)	0.0696 (12)	
C14	1.4005 (5)	0.2414 (4)	0.1802 (4)	0.0677 (11)	
H14	1.4837	0.2182	0.2110	0.081*	
C15	1.2595 (4)	0.3154 (4)	0.2393 (3)	0.0592 (9)	
H15	1.2477	0.3413	0.3109	0.071*	
C16	0.9754 (4)	0.5531 (4)	0.3084 (3)	0.0529 (9)	
H16	1.0474	0.5205	0.3577	0.064*	
C17	0.8230 (4)	0.6224 (4)	0.3794 (3)	0.0574 (9)	
O3	0.7304 (3)	0.7037 (3)	0.3154 (3)	0.0766 (8)	
C18	0.5803 (5)	0.7789 (5)	0.3722 (6)	0.110 (2)	
H18A	0.5602	0.7413	0.4510	0.132*	0.62 (3)
H18B	0.5088	0.7732	0.3345	0.132*	0.62 (3)
H18C	0.5834	0.7912	0.4481	0.132*	0.38 (3)
H18D	0.5213	0.7210	0.3811	0.132*	0.38 (3)
C19	0.571 (2)	0.9256 (10)	0.366 (2)	0.150 (8)	0.62 (3)
H19A	0.6297	0.9317	0.4139	0.226*	0.62 (3)
H19B	0.4690	0.9830	0.3907	0.226*	0.62 (3)
H19C	0.6074	0.9561	0.2886	0.226*	0.62 (3)
C19A	0.500 (3)	0.917 (2)	0.318 (2)	0.140 (12)	0.38 (3)
H19D	0.5704	0.9573	0.2711	0.209*	0.38 (3)
H19E	0.4360	0.9769	0.3760	0.209*	0.38 (3)
H19F	0.4407	0.9071	0.2717	0.209*	0.38 (3)
C20	1.0180 (4)	0.6576 (4)	0.2149 (3)	0.0580 (10)	
O5	1.0258 (4)	0.7581 (3)	0.2602 (3)	0.0796 (9)	
C21	1.0583 (9)	0.8667 (6)	0.1809 (6)	0.131 (3)	
H21A	1.0011	0.8895	0.1224	0.157*	0.48 (3)
H21B	1.1629	0.8346	0.1442	0.157*	0.48 (3)
H21C	1.0677	0.8480	0.1040	0.157*	0.52 (3)
H21D	1.1521	0.8705	0.1864	0.157*	0.52 (3)
C22	1.022 (4)	0.9937 (15)	0.2377 (14)	0.109 (8)	0.48 (3)
H22A	0.9262	1.0138	0.2880	0.164*	0.48 (3)
H22B	1.0221	1.0702	0.1810	0.164*	0.48 (3)
H22C	1.0961	0.9786	0.2806	0.164*	0.48 (3)
C22A	0.937 (2)	1.0027 (12)	0.205 (2)	0.152 (8)	0.52 (3)
H22D	0.8533	1.0101	0.1765	0.228*	0.52 (3)
H22E	0.9727	1.0763	0.1694	0.228*	0.52 (3)
H22F	0.9072	1.0087	0.2862	0.228*	0.52 (3)

### Atomic displacement parameters (Å^2^)

**Table:**

	*U*^11^	*U*^22^	*U*^33^	*U*^12^	*U*^13^	*U*^23^
Se1	0.0812 (4)	0.1012 (4)	0.0863 (4)	−0.0446 (3)	−0.0377 (3)	0.0424 (3)
O1	0.116 (3)	0.095 (3)	0.110 (3)	−0.042 (2)	0.047 (3)	−0.035 (2)
O2	0.067 (2)	0.126 (4)	0.170 (5)	−0.014 (2)	0.018 (3)	−0.035 (3)
O4	0.0721 (19)	0.109 (2)	0.0520 (17)	−0.0186 (18)	0.0079 (14)	−0.0072 (16)
O6	0.125 (3)	0.082 (2)	0.0519 (18)	−0.051 (2)	−0.0073 (17)	0.0087 (14)
N1	0.091 (3)	0.103 (3)	0.085 (3)	−0.051 (2)	−0.023 (2)	0.041 (2)
N2	0.070 (2)	0.082 (2)	0.070 (2)	−0.039 (2)	−0.0106 (18)	0.0203 (19)
N3	0.076 (3)	0.067 (3)	0.115 (4)	−0.029 (2)	0.025 (3)	−0.014 (3)
C1	0.059 (2)	0.060 (2)	0.049 (2)	−0.0287 (18)	−0.0026 (16)	−0.0018 (16)
C2	0.056 (2)	0.054 (2)	0.0456 (19)	−0.0232 (17)	−0.0087 (16)	0.0014 (15)
C3	0.054 (2)	0.058 (2)	0.055 (2)	−0.0286 (18)	−0.0118 (17)	0.0014 (17)
C4	0.061 (3)	0.082 (3)	0.066 (3)	−0.033 (2)	−0.004 (2)	−0.011 (2)
C5	0.055 (3)	0.082 (3)	0.109 (4)	−0.022 (2)	−0.021 (3)	−0.018 (3)
C6	0.080 (3)	0.076 (3)	0.108 (4)	−0.035 (3)	−0.047 (3)	0.015 (3)
C7	0.087 (3)	0.100 (3)	0.068 (3)	−0.052 (3)	−0.032 (2)	0.017 (2)
C8	0.060 (2)	0.080 (3)	0.059 (2)	−0.037 (2)	−0.0125 (19)	0.002 (2)
C9	0.0473 (19)	0.054 (2)	0.0480 (19)	−0.0215 (16)	−0.0091 (15)	0.0014 (15)
C10	0.052 (2)	0.0492 (19)	0.049 (2)	−0.0221 (17)	−0.0031 (16)	−0.0016 (15)
C11	0.065 (2)	0.071 (3)	0.053 (2)	−0.030 (2)	−0.0024 (19)	−0.0099 (19)
C12	0.083 (3)	0.077 (3)	0.061 (2)	−0.038 (3)	0.010 (2)	−0.018 (2)
C13	0.064 (3)	0.051 (2)	0.080 (3)	−0.025 (2)	0.020 (2)	−0.016 (2)
C14	0.053 (2)	0.057 (2)	0.087 (3)	−0.0197 (19)	−0.009 (2)	0.000 (2)
C15	0.054 (2)	0.057 (2)	0.061 (2)	−0.0173 (18)	−0.0054 (18)	−0.0079 (18)
C16	0.052 (2)	0.051 (2)	0.051 (2)	−0.0203 (17)	−0.0052 (16)	0.0026 (16)
C17	0.054 (2)	0.052 (2)	0.061 (2)	−0.0203 (18)	−0.0008 (18)	−0.0071 (18)
O3	0.0510 (16)	0.0754 (19)	0.0778 (19)	−0.0092 (14)	−0.0015 (14)	0.0076 (15)
C18	0.054 (3)	0.088 (4)	0.148 (5)	−0.004 (3)	0.004 (3)	0.003 (4)
C19	0.089 (11)	0.096 (9)	0.207 (18)	0.014 (7)	0.010 (11)	−0.036 (9)
C19A	0.083 (14)	0.159 (19)	0.119 (16)	−0.017 (14)	−0.001 (11)	0.041 (13)
C20	0.050 (2)	0.053 (2)	0.060 (2)	−0.0169 (17)	−0.0017 (17)	0.0025 (18)
O5	0.096 (2)	0.0653 (18)	0.0764 (19)	−0.0450 (17)	0.0138 (16)	−0.0137 (15)
C21	0.165 (6)	0.074 (4)	0.132 (5)	−0.070 (4)	0.049 (5)	−0.015 (3)
C22	0.134 (18)	0.069 (8)	0.116 (11)	−0.042 (9)	−0.006 (10)	0.000 (7)
C22A	0.168 (17)	0.117 (12)	0.181 (18)	−0.080 (13)	−0.056 (15)	0.053 (11)

### Geometric parameters (Å, °)

**Table:**

Se1—C2	1.836 (4)	C14—H14	0.9300
Se1—N1	1.875 (4)	C15—H15	0.9300
O1—N3	1.216 (6)	C16—C17	1.510 (5)
O2—N3	1.208 (7)	C16—C20	1.525 (5)
O4—C17	1.195 (5)	C16—H16	0.9800
O6—C20	1.195 (5)	C17—O3	1.315 (5)
N1—N2	1.260 (5)	O3—C18	1.447 (5)
N2—C1	1.386 (5)	C18—C19	1.4991 (10)
N3—C13	1.472 (6)	C18—C19A	1.4992 (10)
C1—C2	1.361 (5)	C18—H18A	0.9700
C1—C3	1.476 (5)	C18—H18B	0.9700
C2—C9	1.514 (5)	C18—H18C	0.9700
C3—C8	1.379 (5)	C18—H18D	0.9700
C3—C4	1.392 (5)	C19—H19A	0.9600
C4—C5	1.374 (6)	C19—H19B	0.9600
C4—H4	0.9300	C19—H19C	0.9600
C5—C6	1.366 (7)	C19A—H19D	0.9600
C5—H5	0.9300	C19A—H19E	0.9600
C6—C7	1.368 (7)	C19A—H19F	0.9600
C6—H6	0.9300	C20—O5	1.316 (5)
C7—C8	1.380 (6)	O5—C21	1.434 (6)
C7—H7	0.9300	C21—C22	1.4989 (10)
C8—H8	0.9300	C21—C22A	1.4990 (10)
C9—C10	1.517 (5)	C21—H21A	0.9700
C9—C16	1.535 (5)	C21—H21B	0.9700
C9—H9	0.9800	C21—H21C	0.9700
C10—C15	1.378 (5)	C21—H21D	0.9700
C10—C11	1.391 (5)	C22—H22A	0.9600
C11—C12	1.366 (6)	C22—H22B	0.9600
C11—H11	0.9300	C22—H22C	0.9600
C12—C13	1.363 (7)	C22A—H22D	0.9600
C12—H12	0.9300	C22A—H22E	0.9600
C13—C14	1.371 (6)	C22A—H22F	0.9600
C14—C15	1.392 (5)		
			
C2—Se1—N1	87.20 (17)	C17—C16—C9	111.8 (3)
N2—N1—Se1	110.9 (3)	C20—C16—C9	110.1 (3)
N1—N2—C1	117.2 (4)	C17—C16—H16	108.4
O2—N3—O1	123.6 (5)	C20—C16—H16	108.4
O2—N3—C13	117.9 (6)	C9—C16—H16	108.4
O1—N3—C13	118.5 (6)	O4—C17—O3	124.9 (4)
C2—C1—N2	115.9 (4)	O4—C17—C16	123.6 (4)
C2—C1—C3	127.3 (3)	O3—C17—C16	111.5 (3)
N2—C1—C3	116.7 (3)	C17—O3—C18	117.6 (4)
C1—C2—C9	127.6 (3)	O3—C18—C19	106.1 (6)
C1—C2—Se1	108.8 (3)	O3—C18—C19A	118.2 (9)
C9—C2—Se1	123.7 (3)	O3—C18—H18A	110.5
C8—C3—C4	118.5 (4)	C19—C18—H18A	110.5
C8—C3—C1	121.9 (3)	O3—C18—H18B	110.5
C4—C3—C1	119.6 (3)	C19—C18—H18B	110.5
C5—C4—C3	120.7 (4)	H18A—C18—H18B	108.7
C5—C4—H4	119.6	O3—C18—H18C	107.7
C3—C4—H4	119.6	C19A—C18—H18C	107.7
C6—C5—C4	120.2 (4)	O3—C18—H18D	107.7
C6—C5—H5	119.9	C19A—C18—H18D	107.7
C4—C5—H5	119.9	H18C—C18—H18D	107.1
C5—C6—C7	119.5 (5)	C18—C19—H19A	109.5
C5—C6—H6	120.3	C18—C19—H19B	109.5
C7—C6—H6	120.3	C18—C19—H19C	109.5
C6—C7—C8	121.1 (4)	C18—C19A—H19D	109.5
C6—C7—H7	119.4	C18—C19A—H19E	109.5
C8—C7—H7	119.4	H19D—C19A—H19E	109.5
C3—C8—C7	119.8 (4)	C18—C19A—H19F	109.5
C3—C8—H8	120.1	H19D—C19A—H19F	109.5
C7—C8—H8	120.1	H19E—C19A—H19F	109.5
C2—C9—C10	111.3 (3)	O6—C20—O5	124.2 (4)
C2—C9—C16	112.8 (3)	O6—C20—C16	125.6 (4)
C10—C9—C16	112.6 (3)	O5—C20—C16	110.2 (3)
C2—C9—H9	106.5	C20—O5—C21	115.6 (4)
C10—C9—H9	106.5	O5—C21—C22	112.0 (7)
C16—C9—H9	106.5	O5—C21—C22A	110.3 (8)
C15—C10—C11	119.3 (3)	O5—C21—H21A	109.2
C15—C10—C9	121.5 (3)	C22—C21—H21A	109.2
C11—C10—C9	119.1 (3)	O5—C21—H21B	109.2
C12—C11—C10	120.4 (4)	C22—C21—H21B	109.2
C12—C11—H11	119.8	H21A—C21—H21B	107.9
C10—C11—H11	119.8	O5—C21—H21C	109.6
C13—C12—C11	119.4 (4)	C22A—C21—H21C	109.6
C13—C12—H12	120.3	O5—C21—H21D	109.6
C11—C12—H12	120.3	C22A—C21—H21D	109.6
C12—C13—C14	122.2 (4)	H21C—C21—H21D	108.1
C12—C13—N3	119.1 (5)	C21—C22—H22A	109.5
C14—C13—N3	118.7 (5)	C21—C22—H22B	109.5
C13—C14—C15	118.3 (4)	C21—C22—H22C	109.5
C13—C14—H14	120.9	C21—C22A—H22D	109.5
C15—C14—H14	120.9	C21—C22A—H22E	109.5
C10—C15—C14	120.4 (4)	H22D—C22A—H22E	109.5
C10—C15—H15	119.8	C21—C22A—H22F	109.5
C14—C15—H15	119.8	H22D—C22A—H22F	109.5
C17—C16—C20	109.6 (3)	H22E—C22A—H22F	109.5
			
C2—Se1—N1—N2	0.3 (4)	C10—C11—C12—C13	−0.2 (6)
Se1—N1—N2—C1	0.3 (6)	C11—C12—C13—C14	−0.5 (6)
N1—N2—C1—C2	−1.0 (6)	C11—C12—C13—N3	−178.0 (4)
N1—N2—C1—C3	−178.4 (4)	O2—N3—C13—C12	−178.0 (5)
N2—C1—C2—C9	−177.9 (4)	O1—N3—C13—C12	1.8 (6)
C3—C1—C2—C9	−0.8 (6)	O2—N3—C13—C14	4.4 (6)
N2—C1—C2—Se1	1.1 (4)	O1—N3—C13—C14	−175.8 (4)
C3—C1—C2—Se1	178.2 (3)	C12—C13—C14—C15	0.9 (6)
N1—Se1—C2—C1	−0.8 (3)	N3—C13—C14—C15	178.4 (4)
N1—Se1—C2—C9	178.3 (3)	C11—C10—C15—C14	0.1 (5)
C2—C1—C3—C8	50.7 (6)	C9—C10—C15—C14	178.9 (3)
N2—C1—C3—C8	−132.2 (4)	C13—C14—C15—C10	−0.7 (6)
C2—C1—C3—C4	−127.9 (4)	C2—C9—C16—C17	−48.0 (4)
N2—C1—C3—C4	49.2 (5)	C10—C9—C16—C17	−175.1 (3)
C8—C3—C4—C5	−3.1 (6)	C2—C9—C16—C20	−170.0 (3)
C1—C3—C4—C5	175.6 (4)	C10—C9—C16—C20	62.9 (4)
C3—C4—C5—C6	1.7 (7)	C20—C16—C17—O4	−138.2 (4)
C4—C5—C6—C7	0.5 (7)	C9—C16—C17—O4	99.5 (5)
C5—C6—C7—C8	−1.3 (7)	C20—C16—C17—O3	42.6 (4)
C4—C3—C8—C7	2.3 (6)	C9—C16—C17—O3	−79.7 (4)
C1—C3—C8—C7	−176.4 (4)	O4—C17—O3—C18	1.7 (6)
C6—C7—C8—C3	−0.1 (7)	C16—C17—O3—C18	−179.0 (4)
C1—C2—C9—C10	−128.4 (4)	C17—O3—C18—C19	105.5 (15)
Se1—C2—C9—C10	52.8 (4)	C17—O3—C18—C19A	149 (2)
C1—C2—C9—C16	103.8 (4)	C17—C16—C20—O6	−118.3 (4)
Se1—C2—C9—C16	−75.0 (4)	C9—C16—C20—O6	5.1 (6)
C2—C9—C10—C15	−81.3 (4)	C17—C16—C20—O5	62.4 (4)
C16—C9—C10—C15	46.6 (4)	C9—C16—C20—O5	−174.3 (3)
C2—C9—C10—C11	97.5 (4)	O6—C20—O5—C21	3.5 (7)
C16—C9—C10—C11	−134.6 (3)	C16—C20—O5—C21	−177.2 (5)
C15—C10—C11—C12	0.3 (6)	C20—O5—C21—C22	163.5 (15)
C9—C10—C11—C12	−178.5 (4)	C20—O5—C21—C22A	121.8 (17)

### Hydrogen-bond geometry (Å, °)

**Table:**

*D*—H···*A*	*D*—H	H···*A*	*D*···*A*	*D*—H···*A*
C22A—H22D···O2^i^	0.96	2.47	3.38 (4)	159
C11—H11···O6^ii^	0.93	2.56	3.410 (6)	152
C18—H18A···O4	0.97	2.29	2.686 (7)	103
C21—H21C···O6	0.97	2.17	2.627 (6)	107

Symmetry codes: (i) *x*−1, *y*+1, *z*; (ii) −*x*+2, −*y*+1, −*z*.
